# Enantio- and Periselective Nitroalkene Diels-Alder Reactions Catalyzed by Helical-Chiral Hydrogen Bond Donor Catalysts

**DOI:** 10.3390/molecules18089982

**Published:** 2013-08-19

**Authors:** Zhili Peng, Maurice J. Narcis, Norito Takenaka

**Affiliations:** Department of Chemistry, University of Miami, 1301 Memorial Drive, Coral Gables, FL 33146, USA

**Keywords:** asymmetric catalysis, nitroalkene, Diels-Alder reaction, helical chirality

## Abstract

Helical-chiral double hydrogen bond donor catalysts promote the nitroalkene Diels-Alder reaction in an enantio- and periselective manner. This represents the first asymmetric catalytic nitroalkene Diels-Alder reaction via LUMO-lowering catalysis. To gain an insight into this new process, the substrate scope of our catalyst was investigated by exploiting readily available 5-substituted pentamethylcyclopentadienes. The catalyst was found to tolerate dienes with different steric demands as well as dienes substituted with heteroatoms. The synthetic utility of 5-substituted pentamethylcyclopentadienes is rather limited, and thus we have developed a three-step route to 1,4,5,5-tetrasubstituted cyclopentadienes from commercially available ketones.

## 1. Introduction

Asymmetric LUMO-lowering catalysis of the Diels-Alder reaction is one of the most extensively studied methods, and its current state is certainly impressive [1,2,3,4,5,6,7]. However, dienophiles are limited to carbonyl compounds, leaving a tremendous gap in an otherwise robust field of chemistry. The catalytic activation of nitroalkene (a ketene equivalent) for the asymmetric Diels-Alder reaction has remained nonexistent, presumably due to its inherent tendency to undergo the inverse electron-demand hetero Diels-Alder reaction with Lewis acids to provide nitronate products [8,9]. In 2007, we reported that the nitroalkene Diels-Alder reaction can be catalyzed periselectively by double hydrogen bond donors, making the realization of its enantioselective catalysis feasible [10]. It should be mentioned that two different strategies to activate dienes for the nitroalkene Diels-Alder reaction have successfully been developed. Barbas and some other groups have exploited the activated dienes generated *in situ* from enones via enamine catalysis (for selected references, please see [11,12,13,14]). On the other hand, Deng and co-workers successfully developed another strategy, which is the activation of 3-hydroxy-2-pyrones (dienes) via general base catalysis [15].

**Scheme 1:**
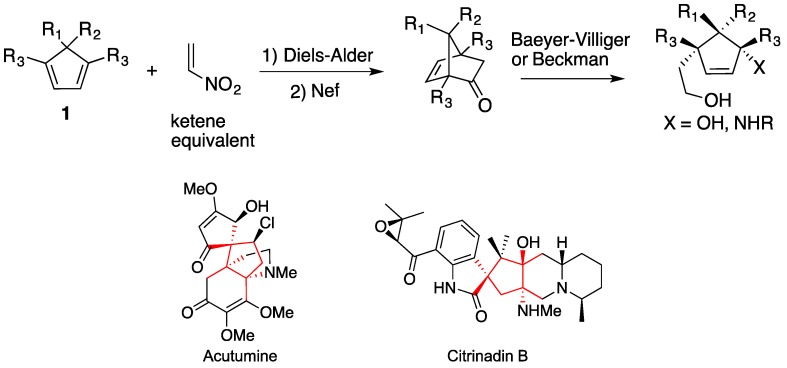
A proven strategy for the synthesis of cyclopentenes.

Our motivation for this study is to fill a gap in the catalytic asymmetric Diels-Alder methodologies that can open ready access to cyclopentenes with multiple contiguous quaternary stereocenters, a motif found in medicinally relevant complex natural products (Scheme 1) [16,17,18].

The Diels-Alder reaction of cyclopentadienes and nitroethylene followed by ring opening reaction is a proven strategy for the synthesis of highly substituted cyclopentenes [19,20]. It should be noted that some research groups have achieved notable advances in the area of asymmetric [3 + 2] reactions (for selected references, please see [21,22,23,24,25,26,27]). However, neither of these [3 + 2] reactions nor the aforementioned nitroalkene Diels-Alder reactions can provide cyclopentenes with three contiguous quaternary stereocenters.

Most of the known hydrogen bond donor catalysts are associated with additional complementary functionalities that activate and constrain an incoming nucleophile to an orientation necessary for asymmetric induction (such bifunctional catalysts typically have the additional hydrogen bond donor or acceptor unit for these purposes) [28,29,30,31]. Obviously, this bifunctional strategy is not applicable for cyclopentadienes, and thus the development of catalysts that do not rely on the additional complementary functionalities is required. We previously designed and developed such catalysts using the addition reaction of pyrroles to nitroalkene as a platform [32,33]. We report herein our studies on the enantio- and periselective nitroalkene Diels-Alder reaction.

## 2. Results and Discussion

### 2.1. Evaluation of Catalysts

**Figure 1:**
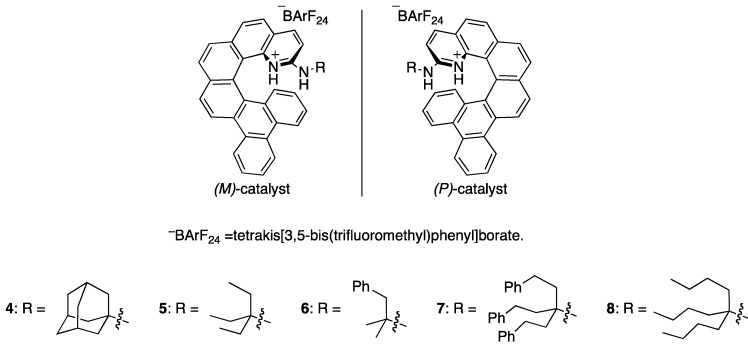
*(M)*- and *(P)* catalysts used.

As part of our broad interest in helicene-derived catalysts, we have recently developed double hydrogen bond donor catalysts based on 1-azahelicene, which do not require additional complementary functionalities for asymmetric induction (Figure 1) [33]. In order to pursue the possibility of catalyzing the nitroalkene Diels-Alder reaction by these type of catalysts, we chose cyclopentadiene **2** as a model substrate because 5-substituted pentamethylcyclopentadienes are readily available [34,35] and are similar to the desired 1,4,5,5-tetrasubstituted cyclopentadienes (**1**) in terms of their structures (Table 1). To our delight, catalyst **4** indeed catalyzed the nitroalkene Diels-Alder reaction, providing only the desired product (*i.e*., no inverse electron-demand hetero Diels-Alder product) with definite enantioselectivity (entry 2). It was somewhat surprising to us that only one diastereomer (compound **3**) was detected by ^1^H-NMR analysis of the crude reaction mixture, although 5,5-disubstituted plane-nonsymmetric cyclopentadienes are known to undergo the Diels-Alder reaction with high π-facial selectivity in accordance with stereocontrol by steric bias [36]. This nitroalkene Diels-Alder reaction was found to proceed with exceedingly high π-facial selectivities for both diene and dienophile (no *exo* isomer either). As demonstrated in our previous study, the catalyst’s enantioselectivity could be tuned by changing the size of the R group that is designed to extend the reach of its helical framework [33]. 

**Table 1:** Evaluation of catalysts for nitroalkene Diels-Alder reaction *^a,b^.* 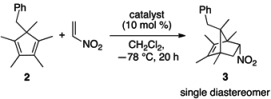

Entry	Catalyst	Yield *^c ^* (%)	er *^d^*
1	-	<5	-
2	(*M*)-**4**	70	64:36
3	(*P*)-**5**	84	38:62
4	(*M*)-**6**	70	59.5:40.5
5	(*M*)-**7**	70	67:33
6	(*M*)-**8**	77	70:30

*^a^* Reaction condition: Diene (0.4 mmol) and nitroethylene (0.2 mmol) in the presence of 10 mol% of catalyst in CH_2_Cl_2_ (0.7 mL); *^b^* These results have been previously communicated, see [34]; *^c^* Yield of isolated products; *^d^* Determined by HPLC analysis.

Accordingly, we examined several R groups, but the enantioselectivity and reactivity of the Diels-Alder reaction turned out only slightly sensitive to the structure of the R substitution (entries 2, 3, 5 and 6). However, catalyst **6**, in which its R group is not evenly substituted, provided distinctly lower enantioselectivity (entry 4). Based on this initial investigation, catalyst **8** seemed optimum.

### 2.2. Evaluation of Dienes

Starting with catalyst **8**, we systematically investigated various 5-substituted pentamethyl-cyclopentadienes. We were glad to find that these dienes were also tolerated by our catalysts (Table 2). The 4-F-substituted diene gave comparable results to a model diene **2**. However, we observed a significantly lower yield with the 4-Cl-substituted diene (entry 3). The reason was not immediately clear to us since all of those dienes are equally reactive under the thermal reaction conditions (*i.e.*, without a catalyst). We hypothesized that there might be steric repulsion between 4-Cl-substitution and the catalyst **8** in the transition state, and thus we tested the sterically less demanding catalyst **4**. We were pleased to find that **4** gave the product in high yield (entry 4).

**Table 2:** Nitroalkene Diels-Alder reaction with different dienes *^a^*^,*b,c*^*.* 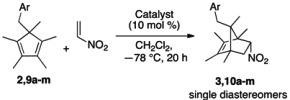

Entry	Diene	Catalyst	Ar	Yield *^d^* (%)	er *^e^*
1	**2**	(*M*)-**8**	Ph	77	70:30
2	**9a**	(*M*)-**8**	4-F-Ph	80	65:35
3	**9b**	(*M*)-**8**	4-Cl-Ph	38	66:34
4	**9b**	(*M*)-**4**	4-Cl-Ph	80	64:36
5	**9c**	(*M*)-**8**	3-Cl-Ph	67	69:31
6	**9d**	(*P*)-**4**	2-Cl-Ph	55	32:68
7	**9e**	(*P*)-**4**	2-I-Ph	65	40:60
8	**9f**	(*P*)-**4**	4-Me-Ph	85	35:65
9	**9g**	(*P*)-**4**	4-CF_3_-Ph	75	36:64
10	**9h**	(*P*)-**4**	4-*tert*-Butyl-Ph	80	30:70
11	**9i**	(*P*)-**4**	2-naphthyl	83	31:69
12	**9j**	(*P*)-**4**	3-OMe-Ph	80	34:66
13	**9k**	(*P*)-**4**	4-SMe-Ph	89	33:67
14 ^*f*^	**9l**	(*P*)-**4**	4-NO_2_-Ph	0	-
15	9m	(P)-4	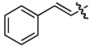	79	37:63

*^a^* Reaction condition: Diene (0.4 mmol) and nitroethylene (0.2 mmol) in the presence of 10 mol % of catalyst in CH_2_Cl_2_ (0.7 mL); *^b^* The absolute stereochemistry of the 4-Cl-substituted product (Entries 3 and 4) was previously established by X-ray analysis [34], the rest were assigned by analogy; *^c^* The results of entries 1 to 5 have been previously communicated, see [34]; *^d^* Yield of isolated products; *^e^* Determined by HPLC analysis; *^f^* Unreacted diene **9l** was recovered (>90%).

The 3- and 2-Cl-substituted dienes and the 2-I-substituted counterpart were also tolerated well by catalyst **4** (entries 5–7). Since we observed a beneficial effect of catalyst **4** with the 4-Cl-substituted diene (entries 3 and 4), we investigated how the size of a 4-substituent would affect the overall reactivity and selectivity of **4**. The 4-Me-substituted diene gave a good yield and moderate selectivity (entry 8). Even the sterically demanding 4-CF_3_-, 4-^t^Bu-substituted and 2-naphthylated dienes were tolerated well by catalyst **4** (entries 9–11).

We were also interested in testing dienes bearing heteroatoms that can function as a hydrogen bond acceptor because such dienes can potentially compete with a dienophile (nitroethylene) for the catalyst’s binding site. We were gratified that the dienes substituted with either O or S atoms were well tolerated by catalyst **4** (entries 12 and 13). Conversely, the diene bearing a nitro group gave no product, an outcome of which might be attributable to the expected similar binding ability of its nitro group to the H-bonding site of the catalyst (entry 14). We were pleased to see the π-facial selectivity of the styrene-substituted diene was also excellent in this reaction albeit its decreased steric bias (entry 15). Only one diastereomer was detected by ^1^H-NMR analysis of the crude reaction mixture.

**Scheme 2:**
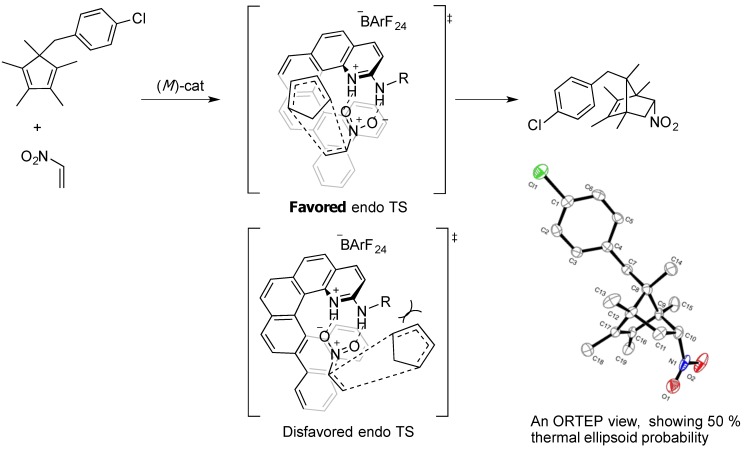
Two *endo* TS models with (*M*)-catalyst. Non-substituted CP is shown for clarity.

Based on the sense of enantioselection observed in the above reactions and the X-ray structure of the HCl salt of catalyst **4** [33], we were able to propose the stereochemical models for the nitroalkene Diels-Alder reaction (Scheme 2). The backside of a bound nitroalkene is completely screened by the bottom half of the helicene framework. In the disfavored transition state, the R group, which is designed to extend the top half of the helical framework, effectively hinders the approach of an incoming diene.

### 2.3. Synthesis of New Dienes

We established that the nitroalkene Diels-Alder reaction can be rendered enantio- and periselective by hydrogen bond donor catalysts. However, the synthetic utility of 5-substituted pentamethyl-cyclopentadienes is rather limited. As such, the 1,4,5,5-tetrasubstituted cyclopentadienes **1** in Scheme 1 would be desired. To our knowledge, the Diels-Alder reaction of such dienes has never been reported (for Diels-Alder reactions of androsta-14, 16-dien-17-yl acetates with nitroethylene, see [37]).

**Scheme 3:**
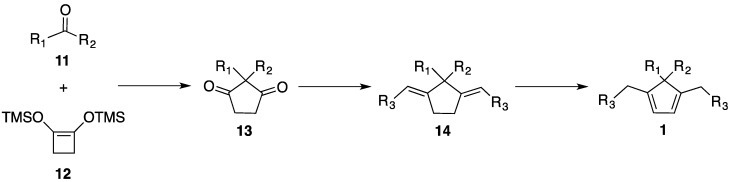
A three-step synthesis of a new class of dienes **1**.

We envisioned that dienes **1** should be accessed by following the synthesis of 5,5-diallyl-1,4-dimethylcyclopenta-1,3-diene reported by Curran and co-worker (Scheme 3) [38]. The synthesis of various diketones **13** from commercially available materials **11** and **12** is well documented by Burnell and co workers [39,40], and thus we began with the alkenation step. Considering the ease of operation and the preparation of ylide, we tested the Wittig reaction first [41]. Unfortunately, the methylenation of diketone **15** by this protocol resulted in no desired product (Table 3, entry 1), presumably because it is sterically congested. We then directed our search to titanium-based alkenating reagents, being non-basic, they have found broad utility for various ketones [42]. Lombardo’s reagent [43,44], which is generated by aging the reaction mixture of CH_2_Br_2_, Zn and TiCl_4_ at 5 °C for 3 days, has been successfully employed for enolizable ketones [45,46]. However, the desired product was formed only in 7% by this protocol (entry 2). Some useful modifications of this method [38,47] (e.g., reagent ratio, reaction time, temperature) reported in the literature were examined, but the difference was marginal (e.g., entry 3). Takai and co-workers [48] have reported a more reactive system made from CH_2_I_2_, Zn and TiCl_4_. Unfortunately, only trace amount of product was observed by TLC analysis (entry 4). A breakthrough was made by applying the methylenation reagent generated from CH_2_Cl_2_, Mg, TiCl_4_ and THF, the method of which was recently developed by Yan and co-workers [49]. We were able to obtain the product in a satisfactory yield (entry 5). The following step was straightforward. The diolefin **16** was cleanly isomerized to **1a** by HI, which can also be done on the crude diolefin with similar efficiency. Thus, we developed an efficient, synthetically versatile preparation of a new class of dienes **1** in three steps, starting from commercially available ketones and 1,2-bis(trimethylsilyloxy)cyclobutene.

**Table 3:** Methylenation of diketone **15** by different protocols. 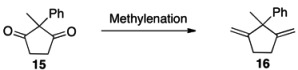

Entry	Protocols [Ref.]	Reagents	Yield *^a^* (%)
1	Wittig [41]	MeP^+^Ph_3_I^−^	0
2	Lombardo [43]	CH_2_Br_2_-Zn-TiCl_4_	7
3	Curran [38]	CH_2_Br_2_-Zn-TiCl_4_	13
4	Takai [48]	CH_2_I_2_-Zn-TiCl_4_	trace
5	Yan [49]	CH_2_Cl_2_-Mg-TiCl_4_-THF	50% *^b^*

*^a^* Yield of isolated product; *^b^* The reaction was not fully optimized.

### 2.4. Evaluation of New Dienes

With the new dienes at hand, we began to investigate their reactivity profiles in nitroalkene Diels-Alder reaction. Oddly enough, the dienes turned out to be quite unreactive toward nitroethylene (Table 4, entries 1, 2 and 4) while analogous 5-substituted pentamethylcyclopentadienes rapidly reacted with nitroethylene under identical conditions. Given the structural similarity between them, such a striking difference in their reactivity is surprising although extra alkyl substituents are expected to raise the HOMO of dienes. Therefore, it was understandable that the H-bond donor catalyst **4** – weakly activating by its very nature – did not promote the reaction (entry 5).

**Table 4:** Evaluation of new dienes. 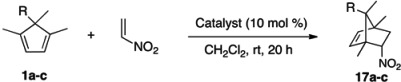

Entry		Catalyst	Yield *^a^* (%)
1	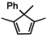	none	0
2	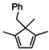	none	25
3		NaBArF_24_∙2.5H_2_O	67 *^b^*
4	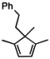	none	11
5		(*P*)-**4**	14 *^c^*
6		NaBArF_24_∙2.5H_2_O	42 *^b^*

*^a^* Yield of isolated product; *^b^* Reactions were not fully optimized; *^c^* 0% ee.

As mentioned in the introduction, there are no obvious ways to activate nitroalkene for the Diels-Alder reaction other than the hydrogen bond donors that are typically weakly activating [8,9,10]. Considering the low reactivity of 1,4,5,5-tetrasubstituted cyclopentadienes toward nitroethylene under thermal conditions, typical hydrogen bond donors do not appear promising. However, our group previously found that the intramolecular nitroalkene Diels-Alder reaction can be catalyzed more efficiently by NaBArF_24_·2.5H_2_O than the typical hydrogen bonding catalysts [50]. To our delight, the NaBArF_24_·2.5H_2_O did catalyze the reaction, and provided the desired products in satisfactory yields (entries 3 and 6), the result of which bodes well for the future endeavor.

## 3. Experimental

### 3.1. General Information

All reactions were carried out in the oven- or flame-dried glassware under an atmosphere of dry argon unless otherwise noted. Except as otherwise indicated, all reactions were magnetically stirred and monitored by analytical thin-layer chromatography using Silicycle pre-coated silica gel plates with F_254_ indicator. Visualization was accomplished by UV light (256 nm), with combination of potassium permanganate and/or vanillin solution as an indicator. Flash column chromatography was performed according to the method of Still using silica gel 60 (mesh 230–400) supplied by Silicycle.

Commercial grade reagents and solvents were used without further purification except as indicated below. Dichloromethane (CH_2_Cl_2_) was freshly distilled over calcium hydride under an atmosphere of dry argon prior to use. THF was freshly distilled over sodium/benzophenone under an atmosphere of dry argon prior to use. Nitroethylene was prepared according to the literature [51].

^1^H-NMR and ^13^C-NMR spectra were recorded on a Bruker Avance 300 (300 MHz ^1^H) and a Bruker Avance 400 (400 MHz ^1^H, 100 MHz ^13^C). Chemical shift values (*δ*) are reported in ppm relative to Me_4_Si (*δ* 0.0 ppm) unless otherwise noted. The proton spectra are reported as follows *δ* (multiplicity, coupling constant *J*, number of protons). Multiplicities are indicated by s (singlet), d (doublet), t (triplet), q (quartet), p (quintet), h (septet), m (multiplet) and br (broad). Optical rotations were measured on a Rudolph Research Analytical AUTOPOL^®^ III automatic polarimeter. Infrared spectra were recorded using PerkinElmer^TM^ SPECTRUM ONE with Universal ATR Sampling Accessory (Composite Zinc Selenide and Diamond crystals). High-resolution mass spectra were obtained at Mass Spectrometry Laboratory, Department of Chemistry, University of Miami.

### 3.2. Preparation of 5-Substituted Pentamethylcyclopentadienes

All the 5-substituted pentamethylcyclopentadienes were synthesized according to our previously reported procedures [34]. The reactions were not optimized.

*1-Chloro-2-((1,2,3,4,5-pentamethylcyclopenta-2,4-dienyl)methyl)benzene* (**9d**). Obtained as colorless oil in 72% yield; ^1^H-NMR (400 MHz, CDCl_3_) *δ* 7.25 (dd, *J* = 1.7, 7.7 Hz, 1H), 6.95–7.04 (m, 2H), 6.78 (dd, *J* = 1.7, 7.6 Hz, 1H), 2.92 (s, 2H), 1.73 (s, 12H), 1.05 (s, 3H); ^13^C NMR (100 MHz, CDCl_3_) *δ* 140.5, 137.0, 134.5, 134.0, 129.6, 128.9, 126.9, 126.1, 56.3, 36.2, 23.4, 11.2, 10.3; FTIR (neat) υ_max_ 2963, 2915, 2859, 1472, 1442, 1379, 1050, 1037, 748 cm^−1^; GCMS: 260[M]^+^.

*1-Iodo-2-((1,2,3,4,5-pentamethylcyclopenta-2,4-dienyl)methyl)benzene* (**9e**). Obtained as a white solid in 61% yield; ^1^H NMR (400 MHz, CDCl_3_) *δ* 7.76 (dd, *J* = 1.2, 7.9 Hz, 1H), 7.04–7.08 (m, 1H), 6.72–6.80 (m, 2H), 2.88 (s, 2H), 1.76 (s, 6H), 1.70 (s, 6H), 1.06 (s, 3H); ^13^C-NMR (100 MHz, CDCl_3_) δ 142.0, 140.6, 139.0, 134.5, 128.4, 127.9, 127.6, 102.5, 56.5, 44,6, 24,1, 11.2, 10.4; FTIR (neat) υ_max_ 2961, 2912, 2858, 1561, 1442, 1378, 1087, 1009, 908, 742 cm^−1^; GCMS: 352[M]^+^

*1-Methyl-4-((1,2,3,4,5-pentamethylcyclopenta-2,4-dienyl)methyl)benzene* (**9f**). Obtained as a white solid in 86% yield; ^1^H-NMR (400 MHz, CDCl_3_) *δ* 6.88 (d, *J* = 7.9 Hz, 2H), 6.74 (d, *J* = 7.9 Hz, 2H), 2.66 (s, 2H), 2.23 (s, 3H), 1.76 (s, 6H), 1.60 (s, 6H), 0.99 (s, 3H); ^13^C-NMR (100 MHz, CDCl_3_) δ 139.6, 135.8, 134.9, 134.5, 128.6, 128.0, 56.9, 41.3, 22.0, 21.1, 11.0, 10.5; FTIR (neat) υ_max_ 2968, 2914, 2857, 1657, 1515, 1442, 1378, 1110, 820, 782, 757 cm^−1^; GCMS: 240 [M]^+^.

*1-((1,2,3,4,5-Pentamethylcyclopenta-2,4-dienyl)methyl)-4-(trifluoromethyl)benzene* (**9g**). Obtained as a white solid in 73% yield; ^1^H-NMR (400 MHz, CDCl_3_) *δ* 7.32 (d, *J* = 8.1 Hz, 2H), 6.92 (d, *J* = 8.1 Hz, 2H), 2.74 (s, 2H), 1.78 (s, 6H), 1.57 (s, 6H), 1.02 (s, 3H); ^13^C-NMR (100 MHz, CDCl_3_) *δ* 142.9, 138.9, 135.2, 128.8, 124.0 (2x), 56.8, 41.3, 21.9, 10.9, 10.4; FTIR (neat) υ_max_ 2968, 2919, 2860, 1618, 1417, 1323, 1162, 1120, 1067, 1019, 847 cm^−1^; GCMS: 294 [M]^+^.

*1-tert-Butyl-4-((1,2,3,4,5-pentamethylcyclopenta-2,4-dienyl)methyl)benzene* (**9h**). Obtained as light yellow oil in 88% yield; ^1^H-NMR (400 MHz, CDCl_3_) *δ* 7.08 (d, *J* = 8.1 Hz, 2H), 6.78 (d, *J* = 8.1 Hz, 2H), 2.66 (s, 2H), 1.76 (s, 6H), 1.59 (s, 6H), 1.25 (s, 9H), 0.99 (s, 3H); ^13^C-NMR (100 MHz, CDCl_3_) δ 148.2, 139.6, 135.7, 134.4, 128.2, 123.9, 56.7, 41.1, 34.3, 31.4, 21.8, 10.9, 10.4; FTIR (neat) υ_max_ 2960, 2913, 2863, 1516, 1448, 1378, 1363, 1269, 1105, 1020, 834, 785 cm^−1^; GCMS: 282 [M]^+^.

*2-((1,2,3,4,5-Pentamethylcyclopenta-2,4-dienyl)methyl)naphthalene* (**9i**). Obtained as a white solid in 81% yield; ^1^H-NMR (400 MHz, CDCl_3_) *δ* 7.70–7.77 (m, 2H), 7.59 (d, *J* = 8.4 Hz, 1H), 7.38–7.42 (m, 2H), 7.36 (s, 1H), 7.03 (d, *J* = 8.4 Hz, 1H), 2.91 (s, 2H), 1.86 (s, 6H), 1.60 (s, 6H), 1.10 (s, 3H); ^13^C-NMR (100 MHz, CDCl_3_) δ 139.5, 136.7, 134.8, 133.3, 132.1, 127.8, 127.7, 127.5, 126.9, 126.4, 125.4, 124.9, 57.0, 41.7, 22.1, 11.0, 10.6; FTIR (neat) υ_max_ 3061, 2965, 2914, 2857, 1601, 1509, 1445, 1378, 1095, 852, 815, 788, 748 cm^−1^; GCMS: 276[M]^+^.

*1-Methoxy-3-((1,2,3,4,5-pentamethylcyclopenta-2,4-dienyl)methyl)benzene* (**9j**). Obtained as a white solid in 84% yield; ^1^H-NMR (400 MHz, CDCl_3_) *δ* 7.02 (t, *J* = 7.8 Hz, 1H), 6.65 (dd, *J* = 2.4, 8.2 Hz, 1H), 6.52 (d, *J* = 7.6 Hz, 1H), 6.45 (s, 1H), 3.72 (s, 3H), 2.72 (s, 2H), 1.80 (s, 6H), 1.65 (s, 6H), 1.02 (s, 3H); ^13^C-NMR (100 MHz, CDCl_3_) δ 158.8, 140.5, 139.6, 134.6, 128.1, 121.4, 113.8, 111.5, 56.8, 55.1, 41.4, 22.2, 11.0, 10.5; FTIR (neat) υ_max_ 2959, 2915, 2858, 1600, 1584, 1490, 1447, 1378, 1291, 1261, 1153, 1048, 910, 776 cm^−1^; GCMS: 256 [M]^+^.

*Methyl(4-((1,2,3,4,5-pentamethylcyclopenta-2,4-dienyl)methyl)phenyl)sulfane* (**9k**). Obtained as a light yellow solid in 87% yield; ^1^H-NMR (400 MHz, CDCl_3_) *δ* 6.99 (d, *J* = 8.3 Hz, 2H), 6.77 (d, *J* = 8.3 Hz, 2H), 2.65 (s, 2H), 2.41 (s, 3H), 1.76 (s, 6H), 1.59 (s, 6H), 0.99 (s, 3H); ^13^C-NMR (100 MHz, CDCl_3_) δ 139.3, 136.0, 134.8, 134.7, 129.2, 126.0, 56.8, 41.1, 21.9, 16.4, 11.0, 10.5; FTIR (neat) υ_max_ 2967, 2916, 2857, 1494, 1441, 1378, 1093, 1017, 835, 810, 781 cm^−1^; GCMS: 272 [M]^+^.

*1-Nitro-4-((1,2,3,4,5-pentamethylcyclopenta-2,4-dienyl)methyl)benzene* (**9l**). Obtained as a bright yellow solid in 10% yield; ^1^H-NMR (400 MHz, CDCl_3_) *δ* 7.93 (d, *J* = 8.8 Hz, 2H), 6.95 (d, *J* = 8.6 Hz, 2H), 2.79 (s, 2H), 1.80 (s, 6H), 1.56 (s, 6H), 1.03 (s, 3H); ^13^C-NMR (100 MHz, CDCl_3_) δ 146.7, 146.3, 138.5, 135.6, 129.2, 122.4, 56.9, 41.3, 21.8, 10.9, 10.5; FTIR (neat) υ_max_ 2918, 2859, 1601, 1516, 1447, 1342, 851, 806, 746,702 cm^−1^; GCMS: 271 [M]^+^.

*(E)-(3-(1,2,3,4,5-Pentamethylcyclopenta-2,4-dienyl)prop-1-enyl)benzene* (**9m**). Obtained as colorless oil in 80% yield; ^1^H-NMR (400 MHz, CDCl_3_) *δ* 7.14-7.28 (m, 5H), 6.30 (d, *J* = 15.8 Hz, 1H), 5.40–5.47 (m, 1H), 2.34 (dd, *J* = 1.4, 7.0 Hz, 2H), 1.79 (s, 6H), 1.76 (s, 6H), 0.97 (s, 3H); ^13^C-NMR (100 MHz, CDCl_3_) δ 140.1, 138.4, 134.2, 129.9, 128.5, 128.1, 126.7, 126.0, 55.9, 38.5, 21.4, 11.2, 10.0; FTIR (neat) υ_max_ 3026, 2962, 2913, 2861, 1600, 1496, 1444, 1378, 960, 738 cm^−1^; GCMS: 252[M]^+^.

### 3.3. General Procedure for Asymmetric Nitroalkene Diels-Alder Reaction

A flame-dried test tube was charged with catalyst **4** (28 mg, 0.02 mmol). To this were added CH_2_Cl_2_ (0.3 mL) and a solution of nitroethylene (15 mg, 0.2 mmol) in CH_2_Cl_2_ (0.1 mL). The resulting mixture was cooled to −78 °C, slowly treated with a solution of hydrazine hydrate (0.1 mL) in MeOH (0.1 mL). The resulting mixture was washed with H_2_O (3 × 1 mL) and brine (1 × 1 mL), dried over Na_2_SO_4_, filtered, and concentrated *in vacuo*. The crude material was purified by flash chromatography on silica gel (2% EtOAc in hexanes). The catalyst ligand was recovered by eluting the column with 100% EtOAc and reused without loss in activity and selectivity.

*(1S,4S,5S,7R)-(−)-7-(2-Chlorobenzyl)-1,2,3,4,7-pentamethyl-5-nitrobicyclo[2.2.1]hept-2-ene* (**10d**). Obtained as a colorless oil in 55% yield with er of 68:32. Enantiomeric ratio was determined by HPLC with a Chiralcel OD-H column equipped with an OD-H guard column (100% hexanes, flow rate = 0.5 mL/min), t_r_ (major) = 54.04 min., t_r_ (minor) = 60.32 min. [α]^20^_D _ = −12, c = 0.0005, CH_2_Cl_2_. ^1^H-NMR (400 MHz, CDCl_3_) δ 7.29–7.31 (m, 1H), 7.09–7.20 (m, 3H), 4.69–4.72 (m, 1H), 2.80 (d, *J* = 14.0 Hz, 1H), 2.67 (d, *J* = 14.0 Hz, 1H), 1.88–1.91 (m, 2H), 1.73 (s, 3H), 1.56 (s, 3H), 1.25 (s, 3H), 0.93 (s, 3H), 0.84 (s, 3H); ^13^C-NMR (100 MHz, CDCl_3_) δ 139.3, 137.8, 135.3, 133.0, 131.3, 129.9, 127.6, 126.2, 91.8, 64.6, 63.9, 56.7, 37.5, 34.9, 15.4, 12.6, 11.5, 11.1, 10.4; FTIR (neat) υ_max_ 2943, 1541, 1444, 1382, 1361, 1058, 877, 753, 682 cm^1^; HRMS (ESI-TOF): Exact mass calcd for C_19_H_24_ClNNaO_2_ [M + Na]^+^, expected: 356.1393, found: 356.1387.

*(1S,4S,5S,7R)-(−)-7-(2-Iodobenzyl)-1,2,3,4,7-pentamethyl-5-nitrobicyclo[2.2.1]hept-2-ene* (**10e**). Obtained as a colorless oil in 65% yield with er of 60:40. Enantiomeric ratio was determined by converting **10e** into **3** via halogen-lithium exchange reaction followed by aqueous work-up, using a Chiralcel OD-H column equipped with an OD-H guard column (100% hexanes, flow rate = 0.5 mL/min), t_r_ (major) = 40.71 min., t_r_ (minor) = 45.09 min. [α]^20^_D _= −16, c = 0.0005, CH_2_Cl_2_. ^1^H-NMR (400 MHz, CDCl_3_) δ 7.79 (d, *J* = 8.0 Hz, 1H), 7.19–7.24 (m, 2H), 6.83–6.85 (m, 1H), 4.68–4.70 (m, 1H), 2.85 (d, *J* = 14.4 Hz, 1H), 2.72 (d, *J* = 14.4 Hz, 1H), 1.88–1.90 (m, 2H), 1.73 (s, 3H), 1.50 (s, 3H), 1.26 (s, 3H), 0.93 (s, 3H), 0.86 (s, 3H); ^13^C-NMR (100 MHz, CDCl_3_) δ 143.2, 140.6, 139.6, 131.9, 131.8, 128.3, 127.9, 103.6, 92.0, 65.0, 64.7, 57.1, 42.3, 37.7, 16.4, 13.5, 12.1, 12.0, 11.0; FTIR (neat) υ_max_ 2943, 1540, 1449, 1382, 1361, 1009, 733, 753 cm^1^; HRMS (ESI-TOF): Exact mass calcd for C_19_H_24_INNaO_2_ [M + Na]^+^, expected: 448.0749, found: 448.0760.

*(1S,4S,5S,7R)-(−)-1,2,3,4,7-Pentamethyl-7-(4-methylbenzyl)-5-nitrobicyclo[2.2.1]hept-2-ene* (**10f**). Obtained as a colorless oil in 85% yield with er of 65:35. Enantiomeric ratio was determined by HPLC with a Chiralcel OD-H column equipped with an OD-H guard column (100% hexanes, flow rate = 0.5 mL/min), t_r_ (major) = 29.73 min., t_r_ (minor) = 33.85 min. [α]^20^_D _= −52, c = 0.0005, CH_2_Cl_2_. ^1^H-NMR (400 MHz, CDCl_3_) δ 7.03 (d, *J* = 7.6 Hz, 2H), 6.94 (d, *J* = 7.6 Hz, 2H), 4.69–4.71 (m, 1H), 2.55 (d, *J* = 13.6 Hz, 1H), 2.45 (d, *J* = 13.6 Hz, 1H), 2.31 (s, 3H), 1.87–1.89 (m, 2H), 1.70 (s, 3H), 1.49 (s, 3H), 1.12 (s, 3H), 0.88 (s, 3H), 0.78 (s, 3H); ^13^C-NMR (100 MHz, CDCl_3_) δ 139.3, 136.3, 135.6, 131.3, 130.7, 128.7, 96.7, 91.8, 64.3, 63.8, 56.2, 21.1, 15.5, 12.6, 11.4, 11.0, 10.3; FTIR (neat) υ_max_ 2936, 1542, 1447, 1362, 1109, 813, 774 cm^−1^; HRMS (ESI-TOF): Exact mass calcd for C_20_H_27_NNaO_2_ [M + Na]^+^, expected: 336.1939, found: 336.1947.

*(1S,4S,5S,7R)-(−)-1,2,3,4,7-Pentamethyl-5-nitro-7-(4-(trifluoromethyl)benzyl)bicyclo[2.2.1]hept-2-ene* (**10g**). Obtained as a colorless oil in 75% yield with er of 64:36. Enantiomeric ratio was determined by HPLC with a Chiralcel OD-H column equipped with an OD-H guard column (100% hexanes, flow rate = 0.5 mL/min), t_r_ (major) = 31.85 min., t_r_ (minor) = 38.25 min. [α]^20^_D_ = −22, c = 0.0005, CH_2_Cl_2_. ^1^H-NMR (400 MHz, CDCl_3_) δ 7.48 (d, *J* = 8.0 Hz, 2H), 7.17 (d, *J* = 8.0 Hz, 2H), 4.69–4.72 (m, 1H), 2.65 (d, *J* = 13.6 Hz, 1H), 2.54 (d, *J* = 13.6 Hz, 1H), 1.87–1.91 (m, 2H), 1.70 (s, 3H), 1.48 (s, 3H), 1.14 (s, 3H), 0.89 (s, 3H), 0.79 (s, 3H); ^13^C-NMR (100 MHz, CDCl_3_) δ 143.9 (d, *J* = 1.4 Hz), 139.3 (d, *J* = 1.5 Hz), 131.5, 131.0, 128.4 (t, *J* = 320.0 Hz), 125.8, 124.9 (dd, *J* = 3.6 Hz, *J* = 72.0 Hz), 91.5, 64.2, 63.9, 56.2, 39.2, 37.6, 15.6, 12.6, 11.4, 11.0, 10.3; FTIR (neat) υ_max_ 2945, 1543, 1323, 1162, 1118, 1067, 1018, 842, 746 cm^1^; HRMS (ESI-TOF): Exact mass calcd for C_20_H_24_F_3_NNaO_2_ [M + Na]^+^, expected: 390.1657, found: 390.1654.

*(1S,4S,5S,7R)-(−)-7-(4-tert-Butylbenzyl)-1,2,3,4,7-pentamethyl-5-nitrobicyclo[2.2.1]hept-2-ene* (**10h**). Obtained as a colorless oil in 80% yield with er of 70:30. Enantiomeric ratio was determined by HPLC with a Chiralcel OD-H column equipped with an OD-H guard column (100% hexanes, flow rate = 0.4 mL/min), t_r_ (major) = 26.76 min., t_r_ (minor) = 29.51 min. [α]^20^_D_ = −54, c = 0.0005, CH_2_Cl_2_. ^1^H-NMR (400 MHz, CDCl_3_) δ 7.23 (d, *J* = 8.4 Hz, 2H), 6.98 (d, *J* = 8.0 Hz, 2H), 4.69–4.72 (m, 1H), 2.55 (d, *J* = 13.6 Hz, 1H), 2.45 (d, *J* = 13.6 Hz, 1H), 1.85–1.89 (m, 2H), 1.70 (s, 3H), 1.48 (s, 3H), 1.3 (s, 9H), 1.12 (s, 3H), 0.88 (s, 3H), 0.78 (s, 3H); ^13^C-NMR (100 MHz, CDCl_3_) δ 149.0, 139.2, 136.3, 131.3, 130.4, 124.8, 91.8, 64.3, 63.8, 56.2, 38.7, 37.7, 34.5, 31.5, 15.6, 12.6, 11.4, 10.9, 10.3; FTIR (neat) υ_max_ 2952, 1542, 1362, 1269, 1110, 908, 835, 731 cm^1^; HRMS (ESI-TOF): Exact mass calcd for C_23_H_33_NNaO_2_ [M + Na]^+^, expected: 378.2409, found: 378.2408.

*(1S,4S,5S,7R)-(−)-2-((1,2,3,4,7-Pentamethyl-5-nitrobicyclo[2.2.1]hept-2-en-7-yl)methyl)naphthalene* (**10i**). Obtained as a colorless oil in 83% yield with er of 69:31. Enantiomeric ratio was determined by HPLC with a Chiralcel AS-H column equipped with an AS-H guard column (100% hexanes, flow rate = 0.5mL/min), t_r_ (minor) = 52.69 min., t_r_ (major) = 69.35 min. [α]^20^_D_ = −48, c = 0.0005, CH_2_Cl_2_. ^1^H-NMR (400 MHz, CDCl_3_) δ 7.73–7.81 (m, 3H), 7.51 (br s, 1H), 7.41–7.48 (m, 2H), 7.21 (dd, *J* = 2.0, 8.4 Hz, 1H), 4.71–4.74 (m, 1H), 2.77 (d, *J* = 13.6 Hz, 1H), 2.61 (d, *J* = 13.6 Hz, 1H), 1.90–1.91 (m, 2H), 1.74 (s, 3H), 1.53 (s, 3H), 1.16 (s, 3H), 0.95 (s, 3H), 0.79 (s, 3H); ^13^C-NMR (100 MHz, CDCl_3_) δ 139.3, 137.1, 133.4, 132.1, 131.4, 129.6, 129.0, 127.7, 127.6, 127.3, 126.1, 125.4, 91.7, 64.3 64.1, 56.3, 39.5, 37.7, 15.7, 12.7, 11.5, 11.0, 10.4; FTIR (neat) υ_max_ 2943, 1540, 1382, 1362, 1084, 821, 748 cm^1^; HRMS (ESI-TOF): Exact mass calcd for C_23_H_28_NO_2_ [M + 1]^+^, expected: 350.2120, found: 350.2116.

*(1S,4S,5S,7R)-(−)-7-(3-Methoxybenzyl)-1,2,3,4,7-pentamethyl-5-nitrobicyclo[2.2.1]hept-2-ene* (**10j**). Obtained as a colorless oil in 80% yield with er of 66:34. Enantiomeric ratio was determined by HPLC with a Chiralcel AS-H column equipped with an AS-H guard column (1% *i*PrOH in hexanes, flow rate = 0.3 mL/min), t_r_ (minor) = 22.48 min., t_r_ (major) = 25.07 min. [α]^20^_D _= −30, c = 0.0005, CH_2_Cl_2_. ^1^H-NMR (400 MHz, CDCl_3_) δ 7.12–7.16 (m, 1H), 6.61–6.73 (m, 2H), 4.68–4.71 (m, 1H), 3.78 (s, 3H), 2.55 (d, *J* = 13.6 Hz, 1H), 2.45 (d, *J* = 13.6 Hz, 1H), 1.88–1.89 (m, 2H), 1.70 (s, 3H), 1.48 (s, 3H), 1.13 (s, 3H), 0.89 (s, 3H), 0.79 (s, 3H); ^13^C-NMR (100 MHz, CDCl_3_) δ 159.3, 141.1, 139.2, 131.3, 128.9, 123.3, 116.8, 111.1, 102.7, 91.7, 64.3, 63.8, 56.2, 55.2, 39.4, 37.7, 15.7, 12.6, 11.5, 10.9, 10.3; FTIR (neat) υ_max_ 2939, 1599, 1583, 1541, 1488, 1362, 1264, 1154, 1045, 768, 699 cm^1^; HRMS (ESI-TOF): Exact mass calcd for C_20_H_27_NNaO_3_ [M + Na]^+^, expected: 352.1889, found: 352.1898.

*(1S,4S,5S,7R)-(−)-Methyl(4-((1,2,3,4,7-pentamethyl-5-nitrobicyclo[2.2.1]hept-2-en-7-yl)methyl)phenyl-)sulfane* (**10k**)*.* Obtained as a colorless oil in 89% yield with er of 67:33. Enantiomeric ratio was determined by HPLC with a Chiralcel AS-H column equipped with an AS-H guard column (1% iPrOH in hexanes, flow rate = 0.3 mL/min), t_r_ (minor) = 25.25 min., t_r_ (major) = 28.71 min. [α]^20^_D _= −60, c = 0.0005, CH_2_Cl_2_. ^1^H-NMR (400 MHz, CDCl_3_) δ 7.13 (d, *J* = 8.4 Hz, 1H), 6.98 (d, *J* = 8.0 Hz, 1H), 4.68–4.71 (m, 1H), 2.42–2.56 (m, 5H), 1.87–1.89 (m, 2H), 1.69 (s, 3H), 1.47 (s, 3H), 1.12 (s, 3H), 0.86 (s, 3H), 0.78 (s, 3H); ^13^C-NMR (100 MHz, CDCl_3_) δ 139.5, 136.7, 136.1, 131.7, 131.6, 126.7, 92.0, 64.5, 64.1, 56.5, 39.1, 38.0, 16.5, 15.9, 13.0, 11.8, 11.3, 10.6; FTIR (neat) υ_max_ 2939, 1540, 1439, 1362, 1090, 810, 755 cm^1^; HRMS (ESI-TOF): Exact mass calcd for C_20_H_28_NO_2_S [M + 1]^+^, expected: 346.1841, found: 346.1845.

*(1S, 4S, 5S, 7R)-(−)-7-Cinnamyl-1,2,3,4,7-pentamethyl-5-nitrobicyclo[2.2.1]hept-2-ene* (**10m**). Obtained as a colorless oil in 79% yield with er of 63:37. Enantiomeric ratio was determined by HPLC with a Chiralcel AS-H column equipped with an AS-H guard column (100% hexanes, flow rate = 0.5mL/min), t_r_ (minor) = 19.25 min., t_r_ (major) = 24.89 min. [α]^20^_D _= −54, c = 0.0005, CH_2_Cl_2_. ^1^H-NMR (400 MHz, CDCl_3_) δ 7.25–7.33 (m, 4H), 7.17–7.23 (m, 1H), 6.03–6.22 (m, 2H), 4.72–4.75 (m, 1H), 2.07–2.19 (m, 2H), 1.89–1.98 (m, 2H), 1.65 (s, 3H), 1.42 (s, 3H), 1.29 (s, 3H), 1.06 (s, 3H), 0.81 (s, 3H); ^13^C-NMR (100 MHz, CDCl_3_) δ 139.3, 137.9, 131.3, 130.3, 128.7, 128.67, 127.0, 126.0, 91.7, 63.9, 63.5, 55.7, 37.4, 36.9, 15.7, 12.7, 11.2, 11.1, 10.2; FTIR (neat) υ_max_ 2939, 1540, 1446, 1362, 1077, 966, 745, 693 cm^1^; HRMS (ESI-TOF): Exact mass calcd for C_21_H_27_NNaO_2_ [M + Na]^+^, expected: 348.1939, found: 348.1931.

### 3.4. New Dienes Synthesis

#### 3.4.1. Synthesis of Diones **13**

All the diones were synthesized according to the reported procedures [39,40].

*2-Methyl-2-phenylcyclopentane- 1,3-dione* (**13a**). Obtained as pale yellow oil in 68% yield. All spectral data were identical to the literature values [52].

*2-Methyl-2-phenemethylcyclopentane-1,3-dione* (**13b**). Obtained as a light orange solid in 72% yield. All spectral data were identical to the literature values [40].

*2-Methyl-2-phenethylcyclopentane-1,3-dione* (**13c**). Obtained as a brown solid in 84% yield; ^1^H-NMR (400 MHz, CDCl_3_) *δ* 7.27–7.07 (m, 5H), 2.58–2.81 (m, 4H), 2.46–2.50 (m, 2H), 1.95–1.99 (m, 2H), 1.16 (s, 3H); ^13^C-NMR (100 MHz, CDCl_3_) δ 216.7, 140.9, 128.9, 128.8, 126.7, 56.8, 37.3, 35.5, 31.2, 20.2; FTIR (neat) υ_max_ 3029, 2922, 1757, 1713, 1497, 1454, 1377, 1269, 1039, 1000, 922, 748, 700 cm^−1^; HRMS (ESI-TOF): Exact mass calcd for C_1__4_H_16_NaO_2_ [M + Na]^+^, expected: 239.1043, found: 239.1043.

#### 3.4.2. General Procedure for the Synthesis of Dienes **1**

The methylenation of diones **13** [49] and the following isomerization of **14** to **1** [38] were carried out with slight modification from the procedures reported in the literature. The reactions were not fully optimized. A typical procedure is as follows: To a mixture of Mg (3.36 g, 140 mmol) and TiCl_4_ (7.65 ml, 70 mmol) in CH_2_Cl_2_(70 mL) cooled down to 0 °C was added via cannula a solution of dione (7.0 mmol) in CH_2_Cl_2_(26 mL) and THF (26 mL). After being stirred for 2 h at 0 °C, the resulting green-black mixture was stirred for another 2 h at room temperature. The reaction mixture was then quenched by slowly adding saturated potassium carbonate solution (150 mL) and diluted with ether (300 mL). The organic layer was then separated, dried, evaporated, and passed through a short plug of silica gel with pentane, and used as crude without further purification in the following step. The crude was then dissolved in PhH (14 ml), and 47% aqueous HI (0.7 ml) was added. The reaction mixture was then stirred vigorously for 20 h in the dark. The reaction was diluted with pentane **(**20 mL), washed with saturated aqueous NaHCO_3_, **(**2 × 15 mL), aqueous Na_2_S_2_O_2_, (2 × 15 mL), and brine **(**15 mL), and the organic phase was dried over a mixture of MgSO_4_ and K_2_CO_3_. The product was purified by flash chromatography (100% hexanes) to yield the desired products.

*(1,2,5-Trimethylcyclopenta-2,4-dienyl)benzene* (**1a**). Obtained as a colorless oil in 25% yield based on dione **13a**; ^1^H-NMR (400 MHz, CDCl_3_) δ 7.25–7.31 (m, 2H), 7.17–7.21 (m, 1H), 7.03–7.06 (m, 2H), 5.98 (s, 2H), 1.70 (s, 6H), 1.38 (s, 3H); ^13^C-NMR (100 MHz, CDCl_3_) δ 154.4 (2x), 128.7, 126.4, 126.3, 125.1, 18.8, 13.3 (2x); FTIR (neat) υ_max_ 3055, 2965, 2932, 2914, 2878, 1600, 1492, 1444, 1372, 1023, 819, 756 cm^−1^; GCMS: 184[M]^+^.

*(2-(1,2,5-Trimethylcyclopenta-2,4-dienyl)methyl)benzene* (**1b**). Obtained as a colorless oil in 28% yield based on dione **13b**; ^1^H-NMR (400 MHz, CDCl_3_) δ 6.96–7.13 (m, 5H), 5.76 (s, 2H), 2.78 (s, 2H), 1.92 (s, 6H), 1.07 (s, 3H); ^13^C-NMR (100 MHz, CDCl_3_) δ 149.4, 138.3, 128.7, 127.6, 126.1, 125.8, 57.2, 41.2, 22.5, 13.4; FTIR (neat) υ_max_ 3046, 2959, 2918, 1604, 1495, 1450, 1377, 1030, 821, 755 cm^−1^; GCMS: 198[M]^+^.

*(2-(1,2,5-Trimethylcyclopenta-2,4-dienyl)ethyl)benzene* (**1c**). Obtained as a colorless oil in 40% yield based on dione **13c**; ^1^H-NMR (400 MHz, CDCl_3_) δ 7.24–7.28 (m, 2H), 7.15–7.19 (m, 1H), 7.11–7.13 (m, 2H), 5.99 (s, 2H), 1.95–1.99 (m, 2H), 1.88 (s, 6H), 1.74–1.78 (m, 2H), 0.99 (s, 3H); ^13^C-NMR (100 MHz, CDCl_3_) δ 149.9, 143,5, 128.6, 128.5, 125.9, 125.5, 56.6, 37.3, 30.5, 22.4, 13.1; FTIR (neat) υ_max_ 3061, 3028, 2965, 2931, 2863, 1604, 1496, 1450, 1376, 1030, 911, 821, 748 cm^−1^; GCMS: 212[M]^+^.

### 3.5. General Procedure for NaBArF_24_∙2.5H_2_O Catalyzed Nitroalkene Diels-Alder Reaction

A flame-dried test tube was charged with NaBArF_24_∙2.5H_2_O (19 mg, 0.02 mmol). To this were added CH_2_Cl_2_ (0.3 mL) and a solution of nitroethylene (28 mg, 0.4 mmol) in CH_2_Cl_2_ (0.1 mL). The resulting mixture was cooled to −78 °C, slowly treated with a solution of diene (0.2 mmol) in CH_2_Cl_2_ (0.3 mL), then the mixture was allowed to warm up to r.t. and stirred for 20 h, and then quenched with a solution of hydrazine hydrate (0.1 mL) in MeOH (0.1 mL). The resulting mixture was washed with H_2_O (3 × 1 mL) and brine (1 × 1 mL), dried over Na_2_SO_4_, filtered, and concentrated *in vacuo*. The crude material was purified by flash chromatography on silica gel (2% EtOAc in hexanes).

*7-Benzyl-1,4,7-trimethyl-5-nitrobicyclo[2.2.1]hept-2-ene* (**17b**). Obtained as a white solid in 67% yield.^ 1^H-NMR (400 MHz, CDCl_3_) δ 7.13–7.26 (m, 5H), 6.20 (d, *J* = 6.0 Hz, 1H), 5.68 (d, *J* = 6.0 Hz, 1H), 4.72–4.75 (m, 1H), 2.80 (d, *J* = 14.4 Hz, 1H), 2.61 (d, *J* = 13.6 Hz, 1H), 2.04–2.10 (m, 1H), 1.92–1.97 (m, 1H), 1.21 (s, 3H), 0.91 (s, 3H), 0.87 (s, 3H); ^13^C-NMR (100 MHz, CDCl_3_) δ 142.1, 139.2, 133.4, 130.8, 128.0, 126.3, 90.3, 65.1, 62.1, 55.5, 39.2, 38.9, 15.1, 14.0, 12.6; FTIR (neat) υ_max_ 2955, 1541, 1452, 1366, 1091, 877, 748, 704 cm^1^; HRMS (ESI-TOF): Exact mass calcd for C_17_H_22_NO_2_ [M + 1]^+^, expected: 272.1651, found: 272.1643.

*1,4,7-Trimethyl-5-nitro-7-phenethylbicyclo[2.2.1]hept-2-ene* (**17c**). Obtained as colorless oil in 42% yield.^ 1^H-NMR (400 MHz, CDCl_3_) δ 7.26–7.29 (m, 2H), 7.12–7.20 (m, 3H), 6.11 (d, *J* = 6.0 Hz, 1H), 5.58 (d, *J* = 6.0 Hz, 1H), 4.78–4.81 (m, 1H), 2.51–2.57 (m, 2H), 2.13–2.18 (m, 1H), 1.96–2.00 (m, 1H), 1.56–1.75 (m, 2H), 1.38 (s, 3H), 1.21 (s, 3H), 0.91 (s, 3H); ^13^C-NMR (100 MHz, CDCl_3_) δ 143.3, 141.7, 133.1, 128.55, 128.2, 125.9, 90.3, 64.1, 61.9, 55.3, 39.0, 35.1, 32.5, 14.7, 14.3, 13.0; FTIR (neat) υ_max_ 2952, 1542, 1453, 1366, 1091, 752, 700 cm^1^; HRMS (ESI-TOF): Exact mass calcd for C_18_H_23_NNaO_2_ [M + Na]^+^, expected: 308.1626, found: 308.1628.

## 4. Conclusions

We successfully demonstrated that the nitroalkene Diels-Alder reaction could be rendered enantio- and periselective by chiral hydrogen bond donor catalysts. This represents, to the best of our knowledge, the first asymmetric catalytic nitroalkene Diels-Alder reaction by LUMO-lowering catalysis. We investigated the substrate scope of catalyst **4** using readily available 5-substituted pentamethylcyclopentadienes. Also, the three-step synthesis of a new class of cyclopentadienes (**1**) was developed, which should find synthetic use.
